# Metal artifact reduction in ^68^Ga-PSMA-11 PET/MRI for prostate cancer patients with hip joint replacement using multiacquisition variable-resonance image combination

**DOI:** 10.1186/s41824-020-00075-x

**Published:** 2020-04-09

**Authors:** Ken Kudura, Tobias Oblasser, Daniela A. Ferraro, Caecilia E. Mader, Lars Husmann, Kerstin Friedrich, Edwin E. G. W. ter Voert, Irene A. Burger

**Affiliations:** 1 Department of Nuclear Medicine, University Hospital Zurich, Zurich, Switzerland; 2 GE Healthcare, Waukesha, WI USA; 3 University of Zurich, Zurich, Switzerland; 4 Department of Nuclear Medicine, Kantonsspital Baden, Baden, Switzerland

## Abstract

**Background:**

PET/MRI has a high potential in oncology imaging, especially for tumor indications where high soft tissue contrast is crucial such as genitourinary tumors. One of the challenges for PET/MRI acquisition is handling of metal implants. In addition to conventional methods, more innovative techniques have been developed to reduce artifacts caused by those implants such as the selective multiacquisition variable-image combination (MAVRIC-SL). The aim of this study is to perform a quantitative and qualitative assessment of metal artifact reduction in ^68^Ga-PSMA-11 PET/MRI for prostate cancer patients with hip joint replacement using a selective MAVRIC-SL sequence for the whole pelvis.

**Methods:**

We retrospectively analyzed data of 20 men with 37 metal hip implants diagnosed with PCA, staged or restaged by ^68^Ga-PSMA-11 PET/MRI from June 2016 to December 2017. Each signal cancellation per side or metal implant was analyzed on the reference sequence LAVA-FLEX, as well as T1-weighted fast spin echo (T1w-FSE) sequence and MAVRIC-SL. Two independent reviewers reported on a four-point scale whether abnormal pelvic ^68^Ga-PSMA-11 uptake could be assigned to an anatomical structure in the tested sequences.

**Results:**

The smallest averaged signal void was observed on MAVRIC-SL sequences with a mean artifact size of 26.17 cm^2^ (range 12.63 to 42.93 cm^2^, *p* < 0.001). The best image quality regarding anatomical assignment of pathological PSMA uptakes in the pelvis by two independent readers was noted for MAVRIC-SL sequences, followed by T1w-FSE with excellent interreader agreement.

**Conclusions:**

MAVRIC-SL sequence allows better image quality in the surrounding of hip implants by reducing MR signal voids and increasing so the accuracy of anatomical assignment of pathological ^68^Ga-PSMA-11 uptake in the pelvis over LAVA-FLEX and T1w-FSE sequences.

## Background

The introduction of combined positron emission tomography and magnetic resonance imaging (PET/MRI) has a high potential in oncology imaging, especially for indications in which good soft tissue contrast is crucial such as genitourinary tumors, head and neck tumors, and liver and breast cancer. The use of this new technology brings up opportunities of new applications for PET imaging such as location of prostate cancer (Eiber et al., 2016a; Pizzuto et al., 2018), assessment of the prostate after focal therapy (Burger et al., 2019), the need of pelvic node dissection (Park et al., 2018) but also technical challenges. One of these challenges is handling of MR metal artifacts for whole body PET/MRI acquisition.

Magnetic resonance imaging (MRI) in the surroundings of metal implants remains challenging (Wollenweber et al., 2013). Metal implants are paramagnetic while human tissue has diamagnetic properties (Landis & Koch, 1977; Kranzbuhler et al., 2018). This different susceptibility leads to local inhomogeneities in the magnetic field, particularly in the vicinity of metal implants (Eiber et al., 2016b).

Metal implants cause significant distortions in the magnetic field, which tends to lead to large artifacts, e.g., signal voids or bright areas on MRI. These artifacts can propagate to MR-based PET attenuation correction (Davidson et al., 2015).

For MRI metal artifact reduction, several conventional methods can lead to a certain reduction, such as using fat saturation via short-tau-inversion-recovery (STIR)- or DIXON-sequences (in-phase, out-phase, fat-/water-attenuation) and fast spin echo sequences instead of gradient echo sequences (Wollenweber et al., 2013). In addition to the conventional methods described above, more innovative techniques have been developed such as the multiacquisition variable-image combination (MAVRIC), first introduced in 2009. MAVRIC is based on a spatially nonselective 3D Fast spin-echo acquisition with changing resonance frequency offsets. MAVRIC-SL adds volume selection to the non-selective MAVRIC technique (Koch et al., 2010). The efficiency of this technique over conventional methods has been demonstrated in several studies (Wengler et al., 2014), most of them using the technique for imaging of metal implants in musculoskeletal imaging. Also for whole-body oncology imaging, the use of MAVRIC-SL has been shown to be beneficial, e.g., for head and neck cancer with reduction of artifacts due to dental implants leading to improvement of attenuation correction (Burger et al., 2015; Gunzinger et al., 2014) and image quality (Abdoli et al., 2016).

Given the significantly higher specificity of the ^68^Ga-labeled ligand targeting the prostate-specific membrane antigen (^68^Ga-PSMA-11) compared to ^11^C- or ^18^F-Choline within the prostate and the superior anatomical allocation of MRI over computer tomography CT (Gunzinger et al., 2014), ^68^Ga-PSMA-11 PET/MRI is a promising new modality for staging high-risk prostate cancer (PCa) with the potential to improve multiparametric MRI (mpMRI) (Eiber et al., 2015; Thalgott et al., 2018). However, especially in this patient population, hip prothesis are present in a substantial number of patients. It is estimated that approximately 1–3% of the adult population over the age of 65 in the USA will require hip arthroplasty during their lifetime (Choi et al., 2015). Therefore, optimal artifact reduction will be crucial in order to improve image quality of ^68^Ga-PSMA-11 PET/MRI in these patients.

The aim of this study is to perform a quantitative and qualitative comparison between conventional methods and MAVRIC-SL sequence for metal artifact reduction in ^68^Ga-PSMA-11 PET/MRI in prostate cancer patients with hip joint replacement.

## Methods

### Patients population

We retrospectively analyzed data of men with uni- or bilateral metal hip implants, compatible with MRI examination, diagnosed with prostate cancer (PCA), staged or restaged by ^68^Ga-PSMA-11 PET/MRI from June 2016 to December 2017. The retrospective use of data required a written informed consent of all included patients following local ethic committee approval (KEK-ZH: 2016-02230).

### ^68^Ga-PSMA-11 PET/MRI acquisition

#### Patient preparation and injection

All included patients were scanned in clinical routine with a 3T wide-bore MR system built on a customized radiofrequency coil with a 25 cm PET detector ring mounted on silicon photomultiplier technology with time of flight PET/MRI system (SIGNA PET/MR; GE Healthcare, Waukesha, WI, USA) (Sekine et al., 2017). Image acquisition started 60 min after intravenous injection of ^68^Ga-PSMA-11 (1.5 MBq/kg). In order to reduce the high ^68^Ga-PSMA-11 activity in the bladder, furosemide (0.13 mg/kg) was injected intravenously 30 min prior to the tracer injection and patients were asked to void prior to the scan.

#### PET acquisition protocol

**Fig. 1:**
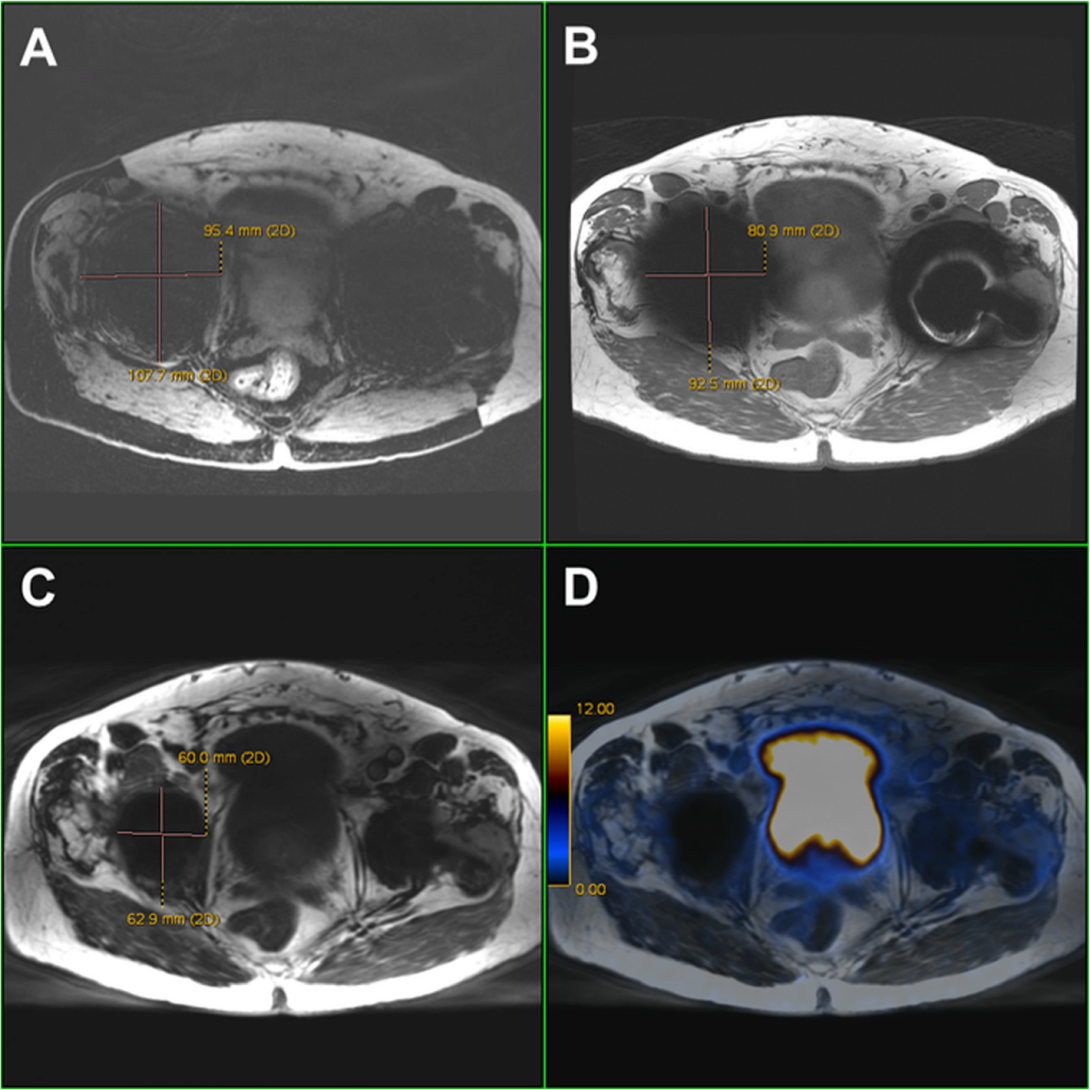
Different appearances of MR signal voids due to hip joint implants. Axial images of a patient with bilateral hip joint implants, referred to ^68^Ga-PSMA-11 PET/MRI for staging prostate cancer. **a** LAVA-FLEX. **b** T1w-FSE. **c** MAVRIC-SL show the different size of signal voids, with the corresponding fused image. **d** PET/MRI MAVRIC-SL

**Fig. 2:**
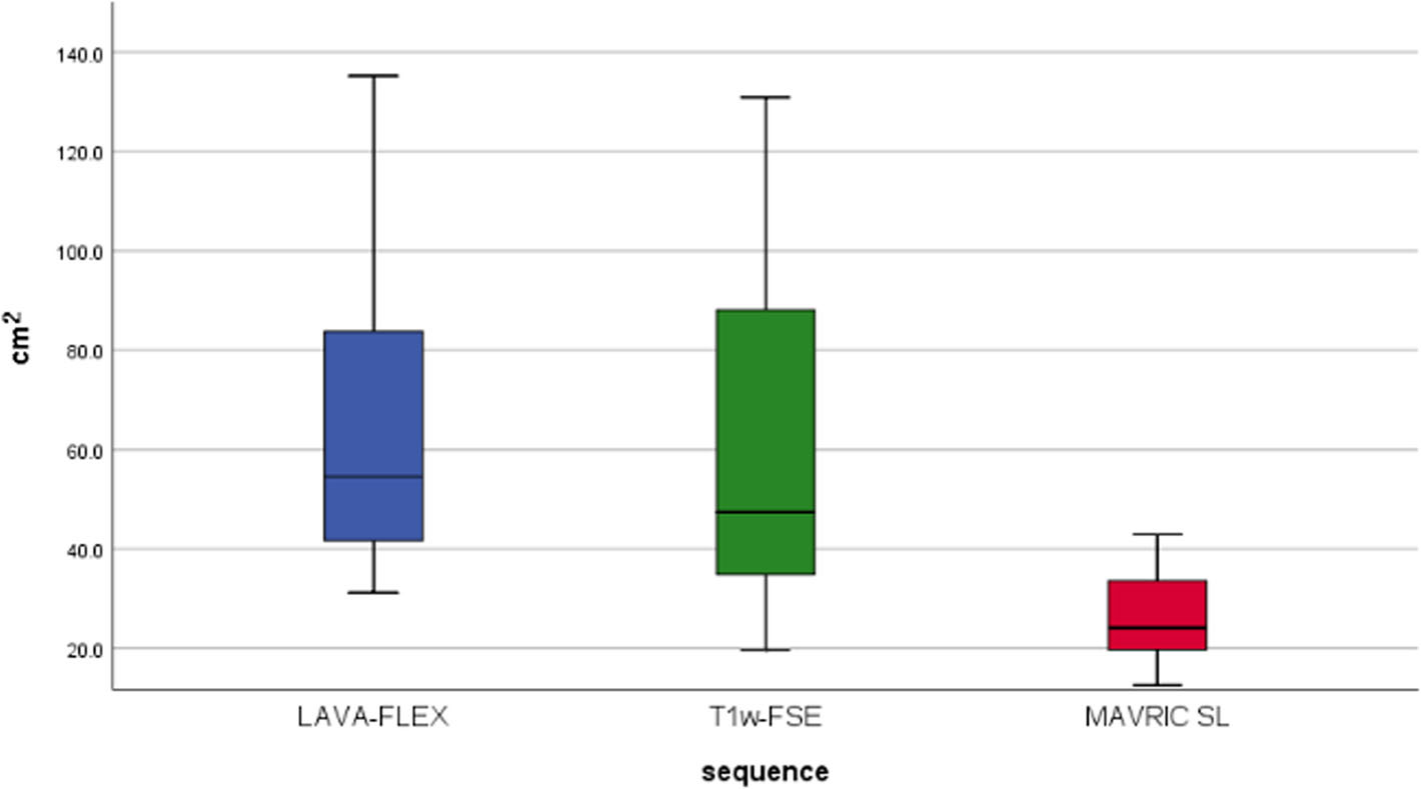
Box plot illustrating the size of signal void (in cm^2^) in the tested sequences

**Fig. 3:**
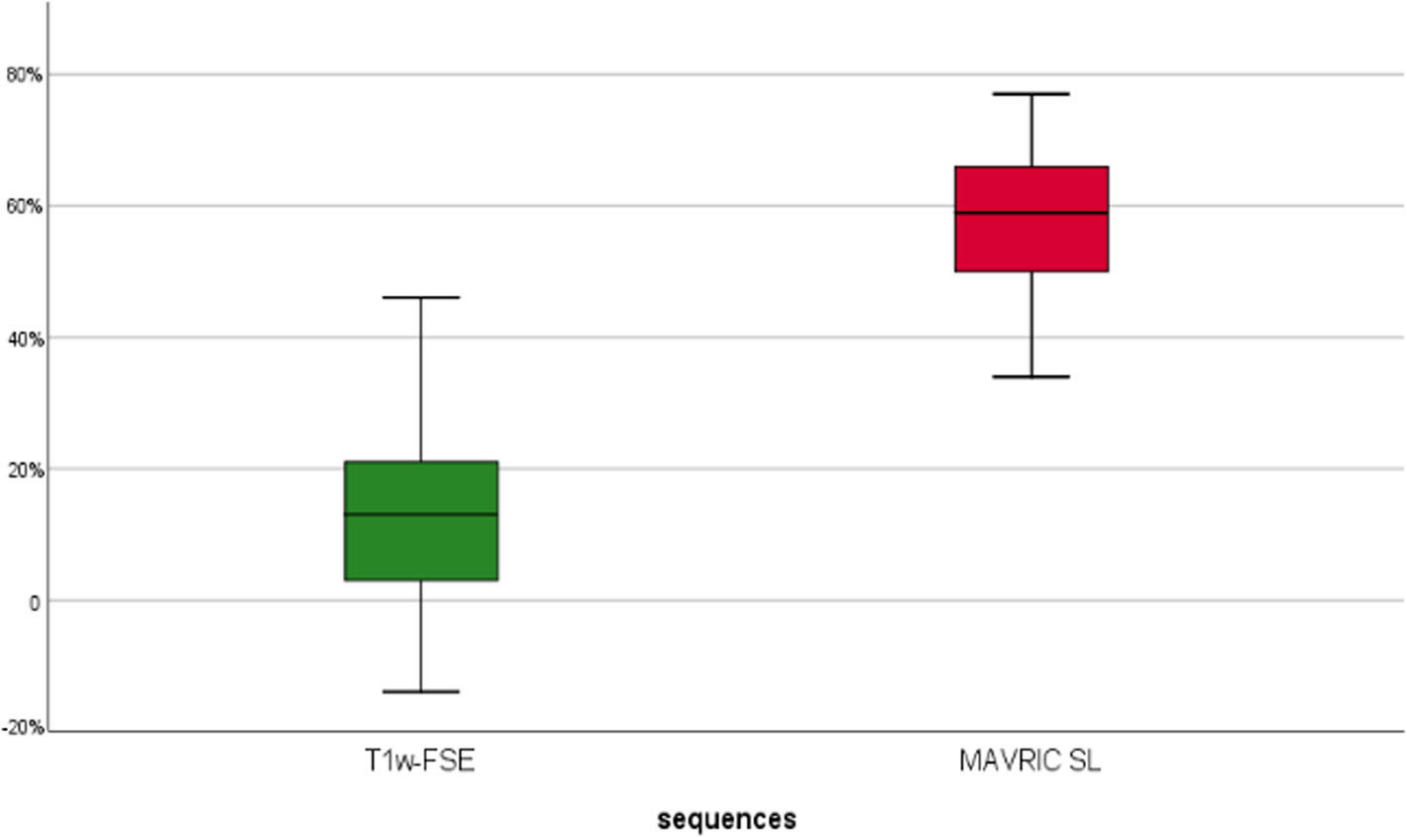
Box plot illustrating the relative signal void reduction (in %) compared to LAVA-FLEX. The relative reduction of signal void was significantly higher (*p* < 0.001%) for MAVRIC-SL with a mean of 57.3% compared to T1w-FSE (9.5%)

**Fig. 4:**
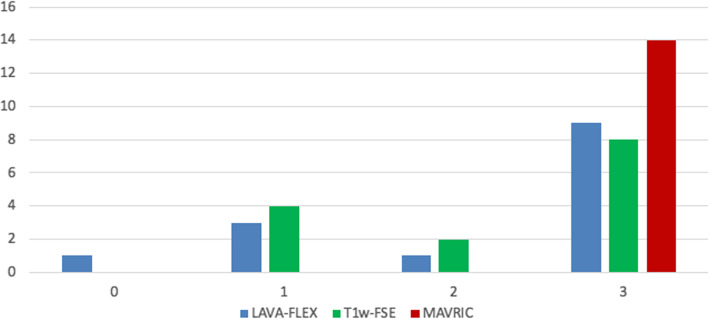
Qualitative assessment of all pathological PSMA uptakes in the pelvis (*n* = 14). 0 = not assessable, 1 = only partially visible, 2 = visible with substantial blurring, 3 = good depiction of anatomical structures. The reported pathological PSMA uptakes in the pelvis could all be assigned to anatomical structures without artifacts in the MAVRIC-SL sequences, e.g., prostate (*n* = 7), lymph node (*n* = 4), or bone structure (*n* = 3)

PET images were acquired with a whole-body protocol from the pelvis to the vertex of the skull with six bed positions and acquisition time of 3 min per bed position, followed by scan of the pelvis for 10 min simultaneous with the acquisition of pelvic T2-weighted fast recovery fast spin echo sequence (FRFSE) in the three planes (Figs. 1, 2, 3, and 4).

#### MRI acquisition protocol

MRI acquisition started with a 3D dual-echo gradient-echo sequence (LAVA-FLEX) commonly used in segmentation of anatomical regions (soft tissue, air, and lung) as reference for whole body MR-based attenuation correction (AC) (Wollenweber et al., 2013). Whole-body acquisition was followed by a 2D T1-weighted fast spin echo (T1w-FSE) sequence and a modified multiacquisition variable-resonance image combination sequence (MAVRIC-SL) over the pelvis. MAVRIC-SL was adjusted from the original release, to enable a large field of view, with one eccentric or even two hip prosthesis. Similar spatial resolution and matrix sizes were used for the three tested sequences (Gunzinger et al., 2014). MR imaging parameters were as described in Table 1.

**Table 1:** Magnetic resonance imaging parameters

MRI parameters	LAVA-FLEX	T1w-FSE	MAVRIC-SL
TR (ms)	5.6	550	959
TE (ms)	2.0	1	6.3
Matrix size	344 × 256	384 × 384	384 × 256
Pixel size (mm)	1.3 × 1.8	0.9 × 0.9	1.3 × 2.0
Slice thickness (mm)	3.2	1.0	5.0
Flip angle (°)	12.0	111.0	85.0
Bandwidth (kHz)	167	63	125

#### PET data reconstruction

All PET data were reconstructed using a Bayesian penalized-likelihood reconstruction algorithm (block sequential regularized expectation maximization, BSREM) with a standard penalty value (*b* value) of 500.

### Quantitative image analysis

For each enrolled patient, we recorded whether a hip implant was unilateral or bilateral. Subsequently, the maximum signal cancellation per metal implant was analyzed, first in the LAVA-FLEX sequence, which was considered as the reference sequence, and afterwards in T1w- FSE and MAVRIC-SL sequences. Quantitative assessment of signal voids was reported for the three tested sequences using commercially available viewing workstations (Advantage Workstation, Version 4.4, GE Healthcare) assuming the shape of the signal cancellation to be elliptical and using the equation *A* = π*(a_1_/2)*(a_2_/2)where *A* is the area of the ellipse, *a*_1_ is the largest diameter, and *a*_2_ is the corresponding orthogonal diameter on the axial images.

### Qualitative image analysis

Images were analyzed in the aforementioned viewing workstations, which enables the review of the ^68^Ga-PSMA-11 PET and MRI images side by side and in fused mode. Two independent reviewers analyzed the images. For each abnormal ^68^Ga-PSMA-11 uptake in the pelvis in the PET images, the reviewers reported whether it could be assigned to an anatomical structure (e.g., prostate lesion, pelvic lymph node, or pelvic bone lesion) on LAVA-FLEX, T1w-FSE, or MAVRIC-SL sequences, using a four-point scale (0 = not assessable, 1 = only partially visible, 2 = visible with substantial blurring, 3 = good depiction of anatomical structures).

### Statistical analysis

The following statistical analyses were performed using SPSS statistics IBM-software version 23.0 (Chicago, IL, USA)**.**

Statistical evaluation of quantitative assessment (e.g., relative and absolute reduction of signal voids for T1w-FSE and MAVRIC-SL sequences compared to the reference sequence LAVA-FLEX) was performed using the Wilcoxon signed-rank test. A *p* value < 0.05 was considered to indicate a significant result.

Statistical evaluation of qualitative assessment (e.g., differences in four-point scale scores between T1w-FSE, MAVRIC-SL, and LAVA-FLEX sequences) was also performed using the Wilcoxon signed-rank test. A *p* value < 0.05 was considered to indicate a significant result.

The interreader agreement was reported using Cohens Kappa, with κ values in the range of 0.81–1 indicating excellent agreement, 0.61–0.80 good agreement, 0.41–0.60 moderate agreement, 0.21–0.40 fair agreement, 0.01–0.2 slight agreement, and 0 indicating poor agreement [2].

## Results

A total of 20 men with 37 hip implants were enrolled. Patient characteristics are given Table 1.

### Quantitative image analysis

The largest averaged signal cancellation was observed on LAVA-FLEX sequences with a mean artifact size of 67.72 cm^2^ (range 31.24 to 154.04 cm^2^), followed by T1w-FSE sequences with a mean artifact size of 61.58 cm^2^ (range 19.69 to 130.80 cm^2^, *p* = 0.003).

The smallest averaged signal cancellation was observed on MAVRIC-SL sequences with a mean artifact size of 26.17 cm^2^ (range 12.63 to 42.93 cm^2^, *p* < 0.001).

Both sequences significantly reduced artifact compared to LAVA-FLEX, with a mean relative artifact reduction of − 9.5% (*p* < 0.001) for T1w-FSE and − 57.3% (*p* < 0.001) for MAVRIC-SL sequences, respectively (Table 2).

**Table 2:** Patients characteristics

Characteristics	Value
Age (years)	
Mean ± SD	72 ± 6.3
Median (range)	73 (59–86)
PSA (ng/ml) at scan (*n* = 19, missing 1)	
Mean ± SD	5.4 ± 7.5
Median	1.86 (0.2–27.3)
0–2.0	11 (58%)
2.1–10	5 (26%)
> 10	3 (16%)
Scan indication (*n* = 20)	
Restaging	15 (75%)
Staging	5 (25%)
Previous prostatectomy (*n* = 20)	
Yes	11 (55%)
No	9 (45%)

### Qualitative image analysis

Both readers independently reported 14 pathological ^68^Ga-PSMA-11 uptakes in the pelvis: seven prostate lesions, four pelvic lymph nodes, and three bone lesions.

Based on a four-point scale (0–3) assessment of whether the abnormal pelvic uptake could be assigned to an anatomical structure, both readers could well-localize pathological ^68^Ga-PSMA-11 uptake within the prostate on all MAVRIC-SL (*n* = 7, mean score 3.00), while slightly impaired localization was observed on LAVA-FLEX (mean score 2.57), and T1w-FSE (mean score 2.29) sequences.

Also pathological nodal ^68^Ga-PSMA-11 uptake (*n* = 4) was best localized on MAVRIC-SL sequences (mean score 3.00). The nodes were only partially seen on T1w-FSE (mean score 2.25), and not detectable on most LAVA-FLEX sequences (mean score 1.75).

Finally, the anatomical correlate for pathological bone ^68^Ga-PSMA-11 uptake in the pelvis (*n* = 3) was only partially visible on LAVA-FLEX (mean score 2.33) and on T1w-FSE (mean score 2.33), but clearly depicted on all MAVRIC-SL sequences (mean score 3.0). Overall, we found an excellent interreader agreement for the evaluation of the correlation between pathological ^68^Ga-PSMA-11 uptake in the pelvis and delineation of anatomical structures for prostatic lesions (κ = 1.000), pelvic lymph nodes (κ = 0.847), and bone metastasis (κ = 0.810) (Table 3).

**Table 3:** Overview of artifact sizes in axial images in the tested sequences

	Minimum	Maximum	Mean	SD
Size of artifact (cm^2^)				
MAVRIC-SL	12.62	42.93	26.17	8.36
T1w-FSE	19.69	130.79	61.57	32.05
LAVA-FLEX	31.23	154.03	67.72	33.70
Relative reduction of artifact (%), compared to LAVA-FLEX				
MAVRIC-SL	+ 23.0	+ 77.0	+ 57.3	+ 12.4
T1w-FSE	− 69.0	+ 46.0	+ 9.5	+ 21.9

## Discussion

Our study shows that MAVRIC-SL sequences can significantly reduce signal voids due to hip implants compared to LAVA-FLEX images and T1w-FSE.

In our quantitative image analysis, the smallest averaged signal cancellation was observed on MAVRIC SL sequences with a significant mean relative artifact reduction of − 57.3% compared to LAVA-FLEX. In other words, signal cancellation due to hip implants on MAVRIC SL was on average more than 50% smaller than on LAVA-FLAX, commonly used.

In our qualitative analysis four ^68^Ga-PSMA-11, uptaking pelvic lymph nodes were on MAVRIC SL morphologically well delineated, on T1w-FSE only partially seen and LAVA-FLEX sequences not detectable. In other words, without a sufficient anatomical assignment of ^68^Ga-PSMA-11 uptake in the pelvis these four uptakes without clear morphological correlation on T1w-FSE and LAVA-FLEX probably would not have been reported as suspicious of lymph node metastasis without MAVRIC SL. Similar observation regarding ^68^Ga-PSMA-11 uptake in pelvic bones whose morphological correlation was on T1w-FSE and LAVA-FLEX partially and on MAVRIC SL totally distinguishable.

In the light of these observations, signal void reduction for pelvic ^68^Ga-PSMA-11 PET/MRI in patients with hip implants can significantly improve anatomical correlation of abnormal ^68^Ga-PSMA-11 uptake in the pelvis using MAVRIC-SL sequences over LAVA-FLEX and T1w-FSE. By improving anatomical assignment MAVRIC SL also improve clinical interpretation of abnormal ^68^Ga-PSMA-11 uptake in the pelvis and so could potentially affect clinical management of prostate cancer patients. However, in order to investigate the impact of MAVRIC SL on clinical management of prostate cancer patients with hip implants, therapy decisions of the interdisciplinary tumorboard based on PET/MRI using MAVRIC SL should be taken into account.

Approximately 1–3% of the adult population over the age of 65 in the USA will require hip arthroplasty during their lifetime (Choi et al., 2015). Therefore, a significant number of patients with prostate cancer will have clinically relevant metal artifacts in the pelvis due to hip implants.

Given that recently published study suggests that ^68^Ga-PSMA-11 PET/MRI may have benefits over PET/CT regarding detection rate of recurrent prostate cancer at very low PSA-levels (Kranzbuhler et al., 2018). Even more evident is the advantage of PET/MR over PET/CT for staging prostate cancer, due to the improved soft tissue contrast on MRI (Eiber et al., 2016b). Therefore, substantial reduction of signal voids seems a promising possibility to improve image quality.

Attenuation correction (AC) remains challenging in combined PET/MRI scanners. The Food and Drug Administration (FDA) approved three methods for attenuation correction (AC) in combined PET/MRI scanners, divided in three main categories: two MR-based with segmentation- (either three- or four-class segmentation) and atlas-based methods and one emission-based (PET-AC) (Izquierdo-Garcia & Catana, 2016). Metal implant-induced signal voids can be corrected by iterative metal artifact reduction algorithms (Schabel et al., 2017; van der Vos et al., 2017) but also semiautomated inpainting artifacts in the pelvis with soft tissue prior to MR-based AC (Torrado-Carvajal et al., 2018), providing so an accurate quantitative activity distribution (Ladefoged et al., 2013).

Our results are in line with the study of Susa et al. suggesting that MAVRIC-SL is able to improve image quality by decreasing signal voids caused by endoprothesis and therefore helping to detect joint abnormalities after joint replacement in patients with musculoskeletal tumors (Susa et al., 2015).

Gutierrez et al. reported MAVRIC-SL sequences improved image quality in the surrounding soft tissue resulting in direct impact on patient management by ruling out the need of further surgery in 13 of 19 patients with implants (Gutierrez et al., 2015).Gunzinger et al. also showed that MAVRIC-SL can be useful to improve diagnostic accuracy in patients with oropharynx tumors by reducing signal voids due to dental implants (Gunzinger et al., 2014).

Limitations of the study are the small cohort of patients and the limited number of ^68^Ga-PSMA-11-positive lesions in the pelvis. Nevertheless, we could already show in this small data set a better anatomical allocation of pathological ^68^Ga-PSMA-11 uptakes in the pelvis to anatomical structures using MAVRIC-SL sequences over LAVA-FLEX and T1w-FSE sequences and a significant reduction of signal void size.

In conclusion, MAVRIC-SL sequences allow better image quality in the surroundings of hip implants by reducing MR signal voids and increasing anatomical assignment of pathological ^68^Ga-PSMA-11 uptake in the prostate, pelvic lymph nodes, and pelvic bones over LAVA-FLEX and T1-weighted FSE sequences.

## Data Availability

All data are available upon reasonable request.

## Abbreviations

^68^Ga-PSMA-11: ^68^Ga labeled ligand targeting the prostate specific membrane antigen
PET/MRI: Positron emission tomography/Magnetic resonance imagingMRI Magnetic resonance imaging
MD: Medical doctor
GE: General electric
STIR: Short-tau-inversion-recovery
MAVRIC: Multi-acquisition variable-image combination
MAVRIC-SL: Multi-acquisition variable-image combination selective
CT: Computerized tomographyPCA Prostate cancer
mpMRI: Multiparametric Magnetic resonance imaging
MBq/kg: Megabecquerel/kilogramFRFSE Fast recovery fast spin echo sequenceAC Attenuation correction
T1w-FSE: T1-weighted fast spin echo
BSREM: Block sequential regularized expectation maximization
PSA: Prostate-specific antigen
FDA: Food and Drug Administration
TR: Repetition time
TE: Echo time
SD: Standard deviation
